# Supplementary material for: Unveiling the hidden depths: advancements in underwater image enhancement using deep learning and auto-encoders
Source: PeerJ Comput Sci. 2024 Nov 29;10:e2392. doi: 10.7717/peerj-cs.2392 (PMC11623242; doi:10.7717/peerj-cs.2392)
Supplement: Supplemental Information 1 [file peerj-cs-10-2392-s001.pdf]

In this work, we have considered two data sets. The dataset links are given below:

Dataset name: EUVP
Dataset Link: https://irvlab.cs.umn.edu/resources/euvp-dataset

Dataset name: UIEB
Dataset Link: https://li-chongyi.github.io/proj_benchmark.html

The proposed work code is available in the supplemental file 2
